# Multiple Logistic Regression Analysis of Risk Factors Associated with Denture Plaque and Staining in Chinese Removable Denture Wearers over 40 Years Old in Xi’an – a Cross-Sectional Study

**DOI:** 10.1371/journal.pone.0087749

**Published:** 2014-02-03

**Authors:** Yanwei Yang, Hongchen Zhang, Zhiguo Chai, Jihua Chen, Shaofeng Zhang

**Affiliations:** 1 Department of Prosthodontics, School of Stomatology, Fourth Military Medical University, Xi’an, China; 2 Department of Clinical Nursing, School of Nursing, Fourth Military Medical University, Xi’an, China; University of North Carolina at Chapel Hill, United States of America

## Abstract

**Background:**

Removable dentures are subject to plaque and/or staining problems. Denture hygiene habits and risk factors differ among countries and regions. The aims of this study were to assess hygiene habits and denture plaque and staining risk factors in Chinese removable denture wearers aged >40 years in Xi’an through multiple logistic regression analysis (MLRA).

**Methods:**

Questionnaires were administered to 222 patients whose removable dentures were examined clinically to assess wear status and levels of plaque and staining. Univariate analyses were performed to identify potential risk factors for denture plaque/staining. MLRA was performed to identify significant risk factors.

**Results:**

Brushing (77.93%) was the most prevalent cleaning method in the present study. Only 16.4% of patients regularly used commercial cleansers. Most (81.08%) patients removed their dentures overnight. MLRA indicated that potential risk factors for denture plaque were the duration of denture use (reference, ≤0.5 years; 2.1–5 years: OR = 4.155, *P = *0.001; >5 years: OR = 7.238, *P*<0.001) and cleaning method (reference, chemical cleanser; running water: OR = 7.081, *P* = 0.010; brushing: OR = 3.567, *P* = 0.005). Potential risk factors for denture staining were female gender (OR = 0.377, *P = *0.013), smoking (OR = 5.471, *P = *0.031), tea consumption (OR = 3.957, *P = *0.002), denture scratching (OR = 4.557, *P* = 0.036), duration of denture use (reference, ≤0.5 years; 2.1–5 years: OR = 7.899, *P = *0.001; >5 years: OR = 27.226, *P*<0.001), and cleaning method (reference, chemical cleanser; running water: OR = 29.184, *P*<0.001; brushing: OR = 4.236, *P* = 0.007).

**Conclusion:**

Denture hygiene habits need further improvement. An understanding of the risk factors for denture plaque and staining may provide the basis for preventive efforts.

## Introduction

Life expectancy has increased in developing and developed countries over the past few decades, and the number and proportion of elderly individuals in the Chinese population have increased steadily. Data from China’s sixth national population census, conducted in 2010, indicate that individuals aged ≥60 years and ≥65 years accounted for 13.26% and 8.87% of the population, respectively. These proportions represent 2.93% and 1.91% increases, respectively, from those in 2000 [1]. With populational aging, the number and proportion of edentulous individuals and those with dental conditions have increased due to factors such as caries and periodontal disease.

China is a developing country, and most Chinese individuals, especially those in the country’s northwestern region, have lower income levels than do people in developed countries. Removable partial and complete dentures remain the primary forms of dental restoration in China because they are inexpensive and have a wide range of indications. Indications for removable partial dentures include long-term edentulism, absence of a posterior abutment for a fixed prosthesis, excessive alveolar bone loss, need for immediate replacement of extracted teeth, and cost considerations. Indications for removable complete dentures include edentulism, inability to preserve remaining teeth, and cost considerations.

Removable dentures are subject to denture plaque and staining for various reasons. Dental and denture plaque can cause or aggravate many oral diseases, such as caries (especially root caries) [2], [3], periodontitis [4], oral candidiasis [5], [6], denture stomatitis [7], [8], and halitosis [9], [10]. Plaque may also serve as a reservoir for pathogens that are significant causes of pneumonia [11] and other systemic diseases [12]. Denture staining is an aesthetic problem that can affect appearance and interpersonal communication [13]–[16].

Through multiple logistic regression analysis (MLRA), Visschere et al. [17] determined that institutional management practices were the only risk factor for denture plaque in patients receiving long-term care in Belgian institutions. Denture staining is reportedly affected by smoking [14], [18], [19], coffee [20], [21] and tea [13]–[15] consumption, duration of denture use [14], and cleaning method [14]–[16]. However, most studies have used monofactorial analyses to investigate factors influencing denture plaque or staining. To our knowledge, few studies have used multivariate analysis to control for confounding factors and evaluate the contributions of multiple factors for denture plaque or staining. MLRA is an effective tool for this purpose.

Paranhos et al. [22] reported that the Budtz-Jorgensen Index is the most appropriate of three denture plaque indices for clinical studies when the use of more sophisticated approaches is not possible. Coulthwaite and Verran [23] reported that blind image scoring was more consistent and repeatable than direct visual assessment. Moreover, digital images can be stored and examined at a later time, enabling longitudinal comparison, remeasurement to assess reproducibility, and double-blind studies [23]. The use of a single examiner enables selection of the most appropriate index for the proposed objective [22]. Thus, the Budtz-Jorgensen Index, blind image scoring, and a single examiner of denture plaque and staining were used in the present study. Prior to the study, the examiner practiced the methods employed, and the criteria and scoring methods were discussed thoroughly [22].

The aims of this cross-sectional study were to investigate hygiene habits and identify risk factors for denture plaque and staining in removable denture wearers aged >40 years in Xi’an using MLRA. Based on our findings, we also provide recommendations for the prevention of denture plaque and staining.

## Materials and Methods

The study sample comprised 222 removable denture wearers who visited the Department of Prosthodontics, Affiliated Stomatology Hospital, Fourth Military Medical University, Xi’an, China, between March and July 2011. This hospital is the premier stomatological hospital in Xi’an, with top-ranking technologies and a large number of patients. The Ethics Committee of Fourth Military Medical University approved the study protocol and all participants provided written informed consent.

Subjects in good general health who wore removable partial or complete dentures (without damage) for at least 1 month were included. Patients with self-reported diabetes, untreated caries, uncontrolled periodontitis, other acute/chronic oral diseases, and/or a history of antibiotic medication use during the last month, and those without the ability to clean dentures daily, were excluded. Voluntary eligible participants were recruited in the clinic’s waiting room.

### Data Collection

One dentist who had been trained at the beginning of the study to ensure consistency conducted structured face-to-face interviews with participants. Subjects were asked to provide information about demographic characteristics (age, gender, educational level, monthly income, smoking habit, tea and coffee consumption), basic denture structure (denture and base types, denture position, attachment use), and daily denture maintenance (duration of denture use, cleaning and overnight storage methods, daily cleaning frequency). A dentist recorded this information using a structured questionnaire based on those used in previous studies [24], [25], with modification of some items to suit the current research purpose. Tables 1 and 2 show how responses to each item were classified. Thereafter, the same dentist clinically examined one denture per participant to assess denture staining, plaque, and surface scratching. If a participant wore maxillary and mandibular dentures, one denture was randomly selected for examination by coin toss.

**Table 1:** Demographic characteristics, basic denture information, and daily maintenance habits of denture wearers in Xi’an, China (*n* = 222).

Parameter	*n*	Plaque score (*n*)		Staining score (*n*)	
		≤Median	>Median	≤Median	>Median
Age (years)					
40–59	45	19	26	21	24
60–79	145	85	60	81	64
≥80	32	19	13	18	14
Total	222	123	99	120	102
Gender					
Male	116	65	51	47	69
Female	106	58	48	73	33
Total	222	123	99	120	102
Educational level					
≤Junior middle school	83	41	42	59	24
Senior middle/vocational school	64	41	23	29	35
≥College	75	41	34	32	43
Total	222	123	99	120	102
Monthly income (Yuan)					
≤1000	33	14	19	24	9
1001–3000	100	60	40	58	42
3001–5000	56	30	26	23	33
>5000	33	19	14	15	18
Total	222	123	99	120	102
Smoking					
No	187	101	86	122	75
Yes	35	22	13	8	27
Total	222	123	99	120	102
Tea consumption					
No	152	84	68	99	53
Yes	70	39	31	21	49
Total	222	123	99	120	102
Coffee consumption					
No	209	113	96	114	95
Yes	13	10	3	6	7
Total	222	123	99	120	102
Denture type					
Maxillary removable partial	70	38	32	42	28
Maxillary complete	42	25	17	26	16
Mandibular removable partial	77	42	35	35	42
Mandibular complete	33	18	15	17	16
Total	222	123	99	120	102
Denture base type					
Plastic	133	70	63	70	63
Metal frame	89	53	36	50	39
Total	222	123	99	120	102
Attachment					
No	209	114	95	112	97
Yes	13	9	4	8	5
Total	222	123	99	120	102
Scratching					
No	28	22	6	24	4
Yes	194	101	93	96	98
Total	222	123	99	120	102
Duration of denture use (years)					
≤0.5	66	52	14	54	12
0.6–2	38	24	14	26	12
2.1–5	48	22	26	20	28
>5	70	25	45	20	50
Total	222	123	99	120	102
Main cleaning method					
Running water	12	5	7	4	8
Brushing	173	89	84	86	87
Chemical cleanser	37	29	8	30	7
Total	222	123	99	120	102
Daily cleaning frequency					
Once	90	50	40	52	38
Twice	84	46	38	45	39
≥3 times	48	27	21	23	25
Total	222	123	99	120	102
Overnight denture removal					
No	42	14	28	11	31
Yes	180	109	71	109	71
Total	222	123	99	120	102

**Table 2:** Relationships between dental plaque and staining scores and study parameters (*n* = 222).

Variable	Plaque score (mean ± SD)	*P*	Staining score (mean ± SD)	*P*
Age (years)				
40–59	1.83±0.48	0.501	1.11±0.74	0.268
60–79	1.72±0.57		0.96±0.52	
≥80	1.71±0.49		0.97±0.55	
Gender				
Male	1.75±0.53	0.844	1.11±0.57	0.001
Female	1.73±0.55		0.85±0.56	
Educational level				
≤Junior middle school	1.77±0.50	0.600	0.89±0.62	0.118
Senior middle/vocational school	1.68±0.54		1.07±0.60	
≥College	1.76±0.58		1.04±0.49	
Monthly income (Yuan)				
≤1000	1.83±0.55	0.428	0.92±0.72	0.455
1001–3000	1.69±0.51		0.96±0.57	
3001–5000	1.80±0.58		1.09±0.49	
>5000	1.72±0.54		0.99±0.60	
Smoking				
No	1.76±0.55	0.285	0.92±0.55	<0.001
Yes	1.65±0.48		1.35±0.58	
Tea consumption				
No	1.75±0.56	0.607	0.88±0.55	<0.001
Yes	1.71±0.48		1.23±0.57	
Coffee consumption				
No	1.75±0.54	0.545	0.99±0.58	0.950
Yes	1.65±0.56		1.00±0.54	
Denture type				
Maxillary removable partial	1.71±0.49	0.309	0.97±0.62	0.590
Maxillary complete	1.63±0.60		0.90±0.56	
Mandibular removable partial	1.82±0.54		1.04±0.56	
Mandibular complete	1.76±0.54		1.05±0.54	
Denture base type				
Plastic	1.79±0.56	0.089	1.05±0.62	0.071
Metal frame	1.67±0.50		0.91±0.50	
Attachment				
No	1.75±0.54	0.384	1.00±0.59	0.241
Yes	1.62±0.48		0.81±0.40	
Scratching				
No	1.45±0.39	<0.001	0.63±0.43	<0.001
Yes	1.78±0.54		1.04±0.58	
Duration of denture use (years)				
≤0.5	1.42±0.39	<0.001	0.64±0.40	<0.001
0.6–2	1.64±0.41		0.83±0.52	
2.1–5	1.92±0.49		1.16±0.58	
>5	1.98±0.58		1.29±0.55	
Main cleaning method				
Running water	2.08±0.58	<0.001	1.40±0.79	<0.001
Brushing	1.78±0.51		1.06±0.54	
Chemical cleanser	1.47±0.56		0.53±0.37	
Daily cleaning frequency				
Once	1.74±0.59	0.968	0.93±0.65	0.407
Twice	1.73±0.49		1.04±0.54	
≥3 times	1.76±0.52		1.02±0.49	
Overnight denture removal				
No	1.92±0.53	0.016	1.42±0.58	<0.001
Yes	1.70±0.53		0.89±0.53	

### Clinical Examination

Before examination, the dentures were rinsed with tap water to remove residual food debris and saliva, and then gently air dried. Dentures were photographed (EOS 50D; Canon Inc., Tokyo, Japan) at a 90° angle from each surface (labial/buccal, lingual/palatal, occlusal/fitting surfaces) of denture bases and artificial teeth with standard film–object distance and exposure time. An assistant ordered the images randomly, and a single blinded examiner visually scored them 1 week after they had been obtained [23]. Staining was assessed visually on the images of six surfaces of each denture (labial or buccal, lingual or palatal, and fitting surfaces of denture base; labial or buccal, lingual or palatal, and occlusal surfaces of artificial tooth) using the Budtz-Jorgensen Index [22] (0, no visual stain; 1, ≤1/3 surface covered with stain; 2, 1/3–2/3 coverage; 3, >2/3 coverage). Total staining scores were calculated by determining the mean of scores for the six sites (maximum score = 3).

For plaque examination, the same procedures used for the assessment of staining were applied after dentures were stained to reveal plaque by immersion for 1 min in 0.25% methylene blue solution (Wolsen Bio-technology Company Limited, Xi’an, China), followed by rinsing for 10 s and drying for 30 s.

Wear status was assessed on all polished denture surfaces under natural light. Scratching was scored as present when at least one scratch was observed on any polished surface.

### Statistical Analysis

Data were analyzed using SPSS software (ver. 17.0 for Windows; SPSS Inc., Chicago, IL, USA). Descriptive statistics were calculated for all variables. Univariate analyses (independent-sample *t*-tests and one-way analysis of variance) were used to assess relationships between the factors surveyed and denture plaque or staining.

MLRA was then performed to identify significant risk factors for denture plaque and staining. MLRA enables quantitative comparison of the separate and joint effects of putative risk factors, with their severity indicated by odds ratios (ORs) [17]. As dependent variables must be categorical, continuous variables must be transformed, usually *via* the classification of quartiles or a value of clinical significance [17], [26]. Thus, the dependent variables in this study (denture plaque and staining scores) were dichotomized using median values as cutoff points (≤median, >median) [17]. Variables found to be significantly correlated with plaque and staining in univariate analyses served as independent variables in the MLRA. After testing for all possible interactions among independent variables to eliminate the influence of confounders, the best fitted logistic regression model was established to determine the possible risk factors for denture plaque or staining. Adjusted ORs and 95% confidence intervals were calculated for all significant variables. *P*<0.05 was considered to be statistically significant.

## Results

The study sample comprised 116 men and 106 women with a mean age of 68.62±10.24 (range, 42–88) years. Table 1 shows the participants’ demographic characteristics, basic denture information, and daily maintenance habits. Brushing was the most prevalent cleaning method (77.93%, 173/222); 16.67% (37/222) of patients regularly used commercial cleansers and 5.41% (12/222) used only running water to clean their dentures. Most (81.08%, 180/222) subjects removed their dentures overnight. The total mean denture plaque score of the 222 participants was 1.74±0.54 and the median score was 1.67. The total mean denture staining score was 0.99±0.58 and the median score was 0.83.

Table 2 shows relationships between denture plaque and staining scores and the variables investigated. Univariate analyses revealed significant correlations between denture plaque scores and wear status, duration of denture use, cleaning habit (all *P*<0.001), and overnight storage method (*P*<0.05). Denture staining scores were correlated significantly with patients’ gender (*P* = 0.001), smoking status, tea consumption, denture wear status, duration of denture use, cleaning habit and overnight storage method (all *P*<0.001).

Denture plaque and staining scores were dichotomized using median values (1.67 and 0.83, respectively) as cutoff points [17]. Omnibus tests of model coefficients indicated that the χ^2^ value of the MLRA model for denture plaque scores was 40.129 and the *P* value was <0.001. MLRA showed that potential risk factors for denture plaque were the duration of denture use (reference, ≤0.5 years; 2.1–5 years: OR = 4.155, *P = *0.001; >5 years: OR = 7.238, *P*<0.001) and cleaning method (reference, chemical cleanser; running water: OR = 7.081, *P* = 0.010; brushing: OR = 3.567, *P* = 0.005; Table 3). Omnibus tests of model coefficients indicated that the χ^2^ value of the MLRA model for denture staining was 115.239 and the *P* value was <0.001. MLRA showed that potential risk factors for denture staining were female gender (OR = 0.377, *P = *0.013), smoking (OR = 5.471, *P = *0.031), tea consumption (OR = 3.957, *P = *0.002), denture scratching (OR = 4.557, *P* = 0.036), duration of denture use (reference, ≤0.5 years; 2.1–5 years: OR = 7.899, *P = *0.001; >5 years: OR = 27.226, *P*<0.001), and cleaning method (reference, chemical cleanser; running water: OR = 29.184, *P*<0.001; brushing: OR = 4.236, *P* = 0.007; Table 4).

**Table 3:** Results of stepwise MLRA with denture plaque as the dependent variable (*n* = 222).

Variable	*P*	OR	95% CI
Duration of denture use (years)	<0.001		
≤0.5	–	1.000	–
0.6–2	0.110	2.087	0.846–5.152
2.1–5	0.001	4.155	1.801–9.590
>5	<0.001	7.238	3.275–15.995
Main cleaning method	0.009		
Running water	0.010	7.081	1.590–31.528
Brushing	0.005	3.567	1.459–8.720
Chemical cleanser	–	1.000	–

Denture plaque scores were dichotomized using the median (1.67) as a cutoff value (≤1.67, >1.67). Independent variables included duration of denture use, denture wear status, and cleaning and overnight storage methods. Omnibus tests of model coefficients indicated that the χ^2^ value of the logistic regression model was 40.129 and the *P* value was <0.001.

**Table 4:** Results of stepwise MLRA with denture staining as the dependent variable (*n* = 222).

Variable	*P*	OR	95% CI
Gender			
Male	–	1.000	–
Female	0.013	0.377	0.174–0.815
Smoking			
No	–	1.000	–
Yes	0.031	5.471	1.169–25.610
Tea consumption			
No	–	1.000	–
Yes	0.002	3.957	1.626–9.631
Scratching			
No	–	1.000	–
Yes	0.036	4.557	1.104–18.814
Duration of denture use (years)	<0.001		
≤0.5	–	1.000	–
0.6–2	0.210	2.311	0.624–8.562
2.1–5	0.001	7.899	2.262–27.581
>5	<0.001	27.226	7.703–96.224
Main cleaning method	0.001		
Running water	<0.001	29.184	4.561–186.745
Brushing	0.007	4.236	1.491–12.036
Chemical cleanser	–	1.000	–

Denture staining scores were dichotomized using the median (0.83) as a cutoff value (≤0.83, >0.83). Independent variables included patients’ gender, smoking status, tea consumption, duration of denture use, denture wear status, and cleaning and overnight storage methods. Omnibus tests of model coefficients indicated that the χ^2^ value of the logistic regression model was 115.239 and the *P* value was <0.001.

## Discussion

Some experimental studies have used more than one operator [27], [28], and McCabe et al. [29] emphasized the importance of calibration in studies involving more than one examiner. However, Davies [30] reported that the fundamental requisite for the adequacy of an index in clinical studies is a high degree of examiner coherence, rendering comparison among examiners unnecessary. The use of a single examiner enables selection of the most appropriate index for the proposed objective [22]. Thus, a single examiner was used in the present study.

According to previous experience and the method of sample estimation for multivariate analyses, the sample size should be at least 5–10 times the number of independent variables [31]. As 15 independent variables were examined in the present study, so the sample of 222 patients was more than adequate.

Brushing with or without toothpaste was the most popular cleaning method in our subjects (77.93%), in agreement with the findings of previous studies conducted in Turkey (44.4–93.6%) [24], [25], [32], [33] and Brazil (98.7–100%) [34], [35], but in contrast to the reported prevalence in England (21.2%) [36]. Cleanser use was the second most popular cleaning method, but its prevalence in our subjects (16.67%) contrasts markedly with that among denture wearers in England (61.3–68%) [36], [37]. This finding highlights the opportunity for the promotion of cleanser use in Xi’an, as this practice is more convenient and efficacious for the removal of biofilm from dentures than is mechanical brushing. Cleaning with running water (5.41%) was the least popular method in this study, whereas Evren et al. [25] reported a 42.8% prevalence (second to brushing) of this practice in Turkish subjects. The proportions of cleaning methods adopted by denture wearers vary widely among countries and regions, likely due to differences in economic development or income levels, oral hygiene education, and individual behavioral habits, among other factors. Because brushing is the most popular cleaning method in Xi’an, dentists in this region should instruct their patients in effective denture brushing, in addition to popularizing the use of denture cleansers.

Most (81.08%) participants in the present study removed their dentures overnight. This practice was much more prevalent in our patients than among those in Brazil (26.3–41.51%) [25], [34], [35] and Turkey (44.8–51.4%) [24], [38]. The greater prevalence of this appropriate habit among denture wearers in Xi’an may reflect regional differences in oral health education from dentists or publically distributed materials. However, its practice should be promoted further through oral health education.

In this study, MLRA was used to identify potential risk factors for denture plaque (duration of denture use and cleaning method) and staining (female gender, smoking, tea consumption, denture scratching, duration of denture use, and cleaning method). Because few studies have reported risk factors for denture plaque or staining, our findings can only be compared with those of univariate analyses.

Through univariate analysis, previous studies have shown that denture plaque, a criterion used for the evaluation of denture cleanliness, was associated with the roughness of denture materials [39], [40], duration of denture use (i.e., age of dentures) [33], [36], [41], cleaning method [41], and overnight denture removal [24]. Our findings are in line with these results. In contrast, Baran and Nalcaci [24] reported that denture plaque was correlated significantly with subjects’ age, gender, educational level, and smoking status, but not with denture age or cleaning method. Kanli et al. [41] found a significant correlation between denture plaque (defined as cleanliness) and the frequency of cleaning, but our analysis did not yield the same results. Similarly, our results differed from those of Visschere et al. [17], who reported that denture plaque was correlated significantly with denture position (maxillary or mandibular). This variability in results may be due to differences in plaque assessment criteria and the characteristics of study subjects, such as age, overall health status, economic and educational levels, cleaning method, overnight denture removal, and region of residence.

Previous univariate analysis have shown that denture staining was associated with smoking [14], [18], [19], tea consumption [13]–[15], duration of denture use [14], and cleaning method [14]–[16]. Our findings are in accordance with these results. Some *in vitro* studies [20], [21] reported that coffee solution caused denture staining, but we found no significant relationship between coffee consumption and denture staining. This difference may be due to the infrequency of coffee consumption among our subjects (13/222). We also identified significant correlations between denture staining and gender, wear status, and overnight storage method. Denture staining scores were significantly higher in men than in women, possibly because smoking and tea consumption were much more prevalent among men (34/116 and 55/116, respectively) than among women (1/106 and 15/106, respectively; both *P*<0.001). Differences in staining between dentures with and without scratches may be attributable to the increased surface roughness caused by scratching, which can facilitate stain retention and increase the difficulty of stain removal. In this study, overnight denture removal significantly reduced denture staining and plaque scores, in accordance with the finding of Baran and Nalcaci [24]. These findings may be explained by the reduction in the duration of denture contact with bacteria and minerals in the oral environment due to overnight removal, which reduces plaque and calculus formation.

Due to the lack of a unified questionnaire assessing denture plaque and staining, the questionnaire used in the present study was compiled on the basis of questionnaire items used in other studies, some of which were modified. All patients included in this study were from the Xi’an region, and can be considered to be representative of populations in northwestern China. Thus, these findings may provide the basis for the prevention of denture plaque and staining and reveal the relative importance of these risk factors in northwestern China. Our results may also provide a reference for the exploration of risk factors for denture plaque and staining in populations in other geographic/cultural regions.

This study had some limitations. It was cross sectional in nature, and the results could be supplemented by a future interventional cohort study. Other possible factors may also be omitted due to our limited cognitive.

## Conclusion

The hygiene habits of denture wearers aged >40 years in the Xi’an region require further improvement. For example, the proportions of patients using denture cleansers and removing their dentures overnight should be increased. An understanding of the risk factors for denture plaque and staining may provide the basis for preventive efforts. Based on the findings of this study, we offer some recommendations to help patients maintain denture cleanliness. Dentists should promote the use of commercial cleansers, as it is the preferred denture cleaning method. Patients should visit their dentists periodically for denture polishing to maintain surface smoothness and replace dentures after long-term use due to plaque and stain accumulation. Patients also should avoid smoking and tea consumption to reduce denture staining.
